# Association between asbestos exposure and pericardial and tunica vaginalis testis malignant mesothelioma: a case–control study and epidemiological remarks

**DOI:** 10.5271/sjweh.3895

**Published:** 2020-10-30

**Authors:** Alessandro Marinaccio, Dario Consonni, Carolina Mensi, Dario Mirabelli, Enrica Migliore, ­Corrado Magnani, Davide Di Marzio, Valerio Gennaro, Guido Mazzoleni, Paolo Girardi, Corrado ­Negro, Antonio Romanelli, Elisabetta Chellini, Iolanda Grappasonni, Gabriella Madeo, Elisa ­Romeo, Valeria Ascoli, Francesco Carrozza, Italo Francesco Angelillo, Domenica Cavone, ­Rosario Tumino, Massimo Melis, Stefania Curti, Giovanni Brandi, Stefano Mattioli, Sergio Iavicoli, ReNaM Working Group

**Affiliations:** 1 Occupational and Environmental Medicine, Epidemiology and Hygiene Department, Italian Workers’ Compensation Authority (INAIL), Roma, Italy; 2 COR Lombardy, Epidemiology Unit, Fondazione IRCCS Ca’ Granda, Ospedale Maggiore Policlinico and University of Milan, Milano, Italy; 3 COR Piedmont, Unit of Cancer Epidemiology, University of Turin and CPO-Piemonte, Torino, Italy; 4 Department of Translational Medicine, University of Eastern Piedmont, Novara, Italy; 5 COR Liguria, UO Epidemiology, IRCCS Az. Ospedaliera Universitaria San Martino, National Cancer Research Institute (IST), Genova, Italy; 6 COR Province of Bolzano, Alto Adige Health Local Unit, Bolzano, Italy; 7 COR Veneto, Occupational Health Unit, Department of Prevention, Padua, Italy; 8 COR Friuli-Venezia Giulia, University of Trieste -Trieste General Hospitals, Clinical Unit of Occupational Medicine, Trieste, Italy; 9 COR Emilia-Romagna, Health Local Unit, Public Health Department, Reggio Emilia, Italy; 10 COR Tuscany, Cancer Prevention and Research Institute, Unit of Environmental and Occupational Epidemiology, Firenze, Italy; 11 COR Marche, University of Camerino, School of Medicinal and Health Products Sciences, Camerino, Italy; 12 COR Umbria, Umbria Region, Department of Prevention, veterinary health and food safety, Perugia, Italy; 13 COR Lazio, Lazio Region, Department of Epidemiology and Department of Radiological, Oncological and Pathological Sciences, Sapienza University, Roma, Italy; 14 COR Molise, Cardarelli Hospital, Oncology Unit, Campobasso, Italy; 15 COR Campania, Second University of Naples, Department of Experimental Medicine, Napoli, Italy; 16 COR Puglia, University of Bari, Department of Interdisciplinary Medicine, Section of Occupational Medicine ‘‘B. Ramazzini’’, Bari, Italy; 17 COR Sicily, Cancer Registry ASP Ragusa and Sicily Regional Epidemiological Observatory, Italy; 18 COR Sardegna, Regional Epidemiological Center, Cagliari, Italy; 19 Department of Experimental, Diagnostic and Specialty Medicine, University of Bologna, Bologna, Italy; 20 Department of Medical and Surgical Sciences, University of Bologna, Bologna, Italy

**Keywords:** epidemiology, Italy, national registry, rare disease

## Abstract

**Objectives:**

The purposes of this study are to describe the epidemiology of pericardial and tunica vaginalis testis mesothelioma and assess the role of asbestos exposure for these rare diseases.

**Methods:**

Based on incident pericardial and tunica vaginalis testis mesothelioma cases collected from the Italian national mesothelioma registry (ReNaM) in the period 1993–2015, incidence rates, survival median period and prognostic factors have been evaluated. A case–control study has been performed to analyze the association with asbestos exposure (occupational and non-occupational) for these diseases.

**Results:**

Between 1993 and 2015, 58 pericardial (20 women and 38 men) and 80 tunica vaginalis testis mesothelioma cases have been registered with a mean annual standardized (world standard population as reference) incidence rates of 0.049 (per million) in men and 0.023 in women for the pericardial site, and 0.095 for tunica vaginalis testis mesothelioma. Occupational exposure to asbestos was significantly associated with the risk of the diseases [odds ratio (OR) 3.68, 95% confidence interval (CI) 1.85–7.31 and OR 3.42, 95% CI 1.93–6.04 in pericardial and tunica vaginalis testis mesothelioma, respectively]. The median survival was 2.5 months for pericardial and 33.0 months for tunica vaginalis testis mesotheliomas. Age was the main predictive factor for survival for both anatomical sites.

**Conclusions:**

For the first time in an analytical study, asbestos exposure was associated with pericardial and tunica vaginalis testis mesothelioma risk, supporting the causal role of asbestos for all anatomical sites. The extreme rarity of the diseases, the poor survival and the prognostic role of age have been confirmed based on population and nationwide mesothelioma registry data.

Malignant mesothelioma (MM) is a neoplasm of mesothelial cells, and the International Agency for Research on Cancer (IARC) has firmly established the causal role of asbestos (1–3). Despite international health institutes’ and agencies’ recommendations (4–7), currently 2 000 000 tons of asbestos per year are still produced worldwide (8, 9). Pericardial and tunica vaginalis of testis (TVT) MM accounted for around 1% of cases in the available MM case series (10–13). Incidence and survival for these forms have seldom been reported. The surveillance, epidemiology, and end results (SEER) program from the US National Cancer Institute provided evidence of mean annual standardized incidence rates, in 1973–2013, for pericardial MM of 0.35 and 0.36 (per 10 million person-years) in men and women, respectively, and of 0.54 for TVT MM (14).

The causal role of asbestos exposure in pericardial and TVT MM aetiology has been considered as plausible, but no epidemiological analytical study ever tested the asbestos (or other putative risk factors) role for these diseases (11, 12, 15, 16). Italy is one of the countries more widely involved in the current epidemic of asbestos-related diseases due to the large use of asbestos in the past and the number of exposed individuals among workers (and in the general population) until the asbestos ban issued in 1992 (17–20). In this context, a permanent and mandatory epidemiologic surveillance system on MM is active, based on a national MM registry (Registro Nazionale dei Mesoteliomi, ReNaM in Italian). ReNaM aims are to provide estimates of MM incidence at national population level to assess and record asbestos exposures of cases and identify any possible underestimated or unknown source of asbestos contamination.

This study aims to (i) describe incidence and survival for pericardial and TVT MM cases detected by ReNaM and (ii) assess the association with asbestos exposure using a case-control design.

## Methods

### Incidence and survival analyses

ReNaM is an epidemiological surveillance system characterized by a network of regional operating centers [Centri Operativi Regionali (COR) in Italian] gradually established by all 20 Italian regions. Case series from Calabria, Sardinia and Molise are available but these regions still cannot ensure completeness of registration. Reporting is compulsory, but COR also actively search and register incident MM cases at the healthcare services that diagnose and treat most cases (pneumology and chest surgery units as well as pathology units). Completeness of registration is periodically checked by surveys, using regional current health sources, pathology units, hospital admissions and mortality registries. According to ReNaM guidelines (21) and as extensively described elsewhere (22), cases are classified as “definite” (histological confirmation of diagnosis, possibly completed by immunohistochemical characterization, and confirmation by imaging and clinical diagnosis), “probable” (usually, cytological diagnosis and confirmation by imaging and clinical diagnosis) or “possible” MM (clinical diagnosis with positive imaging). The occupational and residential history and information on lifestyle habits are obtained using a standardized questionnaire administered by trained interviewers to cases or their next of kin. COR assess both occupational and non-occupational exposure to asbestos. Occupational histories are coded using the Italian standard classifications of industry and occupation. In each COR, experts carry out the exposure assessment in cooperation, if necessary, with the industrial health and safety units of local health authorities. Lifetime asbestos exposure is classified as “occupational” (definite, probable, possible) or “non-occupational” (familial, environmental, other non-occupational – such as leisure-time-related activities), according to the ReNaM guidelines (21). “Unlikely” exposure is assigned to subjects for whom information is inadequate or asbestos exposure could be reasonably ruled out.

Actually, ReNaM has collected cases with a diagnosis of MM from 1993–2015. Pericardial and TVT MM cases were extracted and analyzed for the whole period. Age and gender standardized incident rates have been calculated using the world, European (as proposed by Eurostat in 2013) and Italian (2011 census) populations as standard populations. The distribution and the extent of person-years of observations is reported in the supplementary material (www.sjweh.fi/show_abstract.php?abstract_id=3895) table S1. Survival was estimated by the Kaplan-Meier method, and Cox’s proportional hazard regression has been used to assess the role of prognostic factors separately for pericardial and TVT MM. The predictive variables in the final Cox’s multivariate model were gender (men and women), age at diagnosis (categorized as follows: ≤64, 65–74, ≥75 years old), calendar period of diagnosis (1993–2003 and 2004–2015), diagnosis level of certainty (definite, probable and possible MM) and morphology (epithelioid, biphasic, fibrous, unspecified MM). The reference modality was the first for all predictive variables in the model. The model’s goodness-of-fit to empirical data was assessed by the log likelihood test.

### Case–control study

We conducted a case–control study using (i) pericardial and TVT MM cases registered by ReNaM in the period 1993–2015, during which coverage of the Italian population progressed as previously described, and (ii) two sets of controls recruited in two earlier case–control studies: a population-based study on pleural mesothelioma and a hospital-based study on cholangiocarcinoma.

We only used controls who had completed the questionnaire. The distribution of controls by gender, age, year of birth, and region of residence is described in table S3. The first set of controls was taken from a multicentric unpublished population-based case–control study on pleural mesothelioma (called MISEM), performed in five Italian regions (Apulia, Lombardy, Piedmont, Tuscany, and Veneto). Controls were frequency-matched to cases by gender and age and randomly sampled from residents aged 20–89 years in 2012 (Apulia, Piedmont, Tuscany and Veneto) and 2014 (Lombardy). Interviews were performed in 2014–2015 and the participation rate was 48.4%. The second set of controls was taken from a hospital-based unpublished case–control study on cholangiocarcinoma performed in Emilia-Romagna region (called CARA) in which controls were enrolled and interviewed in 2014–2016, and that was an evolution of a previous epidemiological study (23). Participation rate was almost complete. Both sets of controls were interviewed using the same standardized ReNaM questionnaire and assessment of asbestos exposure was performed following ReNaM guidelines.

We calculated odds ratios (OR) and 95% confidence intervals (CI) of pericardial and TVT MM for lifetime asbestos exposure (occupational, familial, environmental, and leisure activity related) using “unlikely” asbestos exposure as reference. To avoid sparse data problems, we fitted conditional regression models using age categories (<55, 55–59, 60–64, 65–69, 70–74, 75–79, and ≥80 years) as the adjustment set (24). For pericardial MM, we calculated gender-specific and -adjusted analyses. Sensitivity analyses were performed by applying the following restrictions to cases: (i) only cases from the six regions which enrolled control subjects (table S4); (ii) only cases with definite diagnosis (table S5); (iii) only cases diagnosed in 2000–2015 (table S6); 4) only subjects born before year 1950 (table S7). Finally, we performed specific analyses by economic sectors of exposure. Statistical analyses were performed with Stata version 15, 2017 (StataCorp, College Station, TX, USA).

## Results

### Incidence and survival analyses

Between 1993 and 2015, 58 pericardial MM cases (38 and 20 in men and women, respectively) and 80 TVT MM were registered in ReNaM (table 1). The mean age at diagnosis was 61.8 [standard error (SE) 2.4] years and 61.4 (SE 3.1) in men and women, respectively, for pericardial MM and 66.7 (1.8) for TVT MM. The gender ratio for pericardial cases (male versus female) was 1.95, ranging from 1.5 in 1993–2003 to 2.6 in 2004–2015. The majority of cases were born before year 1950 in both sites and genders. Histological confirmation was obtained for 87.7% of cases; for 17 cases, only cytology or positive imaging were available. The epithelioid form is predominant in both anatomical sites (37.9% in pericardial cases and 52.5% in testicular cases). Incidence analyses, reported in supplementary table S2, confirm the extreme rarity of these diseases. Mean annual standardized incidence rates for pericardial MM in the overall period 1993–2015 were 0.080 (× 1 000 000 inhabitants) in men and 0.036 in women, using the European standard population as reference (0.049 and 0.023 if world population was used). Pericardial MM incidence rates were higher in 1998–2003 for both genders (0.067 and 0.039 in men and women, respectively, with world population standardization), but no reliable temporal trend analysis was possible. TVT MM mean annual incidence rates were 0.095, with the peak in the same period (0.116 in 1998–2003).

**Table 1:** Pericardial and tunica vaginalis testis mesothelioma cases by gender, age at diagnosis, periodof incidence, diagnostic certainty, morphology and asbestos exposure. Italian national mesothelioma registry (ReNaM), 1993–2015. [MM=malignant mesothelioma; NOS=not otherwise specified.]

	Pericardial MM			Tunica vaginalis testis MM		
	
	Women	%	Men	%	N	%
Age classes (years)						
0–44	2	10.0	6	15.8	10	12.5
45–64	8	40.0	9	23.7	15	18.8
65–74	7	35.0	15	39.5	25	31.3
≥75	3	15.0	8	21.2	30	37.5
Period of diagnosis						
1993–1997	3	15.0	5	13.2	8	10.0
1998–2003	9	45.0	12	31.6	23	28.8
2004–2009	2	10.0	11	28.9	24	30.0
2010–2015	6	30.0	10	26.3	25	31.3
Year of birth						
1914–1930	5	25.0	8	21.0	26	32.5
1930–1939	6	39.0	11	29.0	23	28.7
1940–1949	3	15.0	7	18.4	10	12.5
1950–1959	1	5.0	6	15.8	11	13.8
1960–1992	5	25.0	6	15.8	10	12.5
Diagnostic certainty						
MM definite	15	75.0	30	78.9	76	95.0
MM probable or possible	5	25.0	8	21.1	4	5.0
Morphology				
Epithelioid	8	40.0	13	34.2	42	52.5
Biphasic	4	20.0	5	13.2	12	15.0
Sarcomatoid	2	10.0	4	10.5	5	6.3
MM NOS	3	15.0	14	36.8	21	26.3
Not available	3	15.0	2	5.3	-	-
Follow up						
Death	20	95.0	36	94.7	47	58.8
Live at follow up	-	-	2	5.3	33	41.3
Exposure detection						
Indirect interview	14	70.0	20	52.6	23	28.8
Direct interview	3	15.0	8	21.1	45	56.3
No exposure assessment	3	15.0	10	26.3	12	15.0
Modalities of asbestos ­exposure (only for cases with exposure assessment)						
Occupational (definite)	-	-	8	28.6	25	36.8
Occupational (probable)	1	5.9	4	14.3	5	7.4
Occupational (possible)	3	17.6	9	32.1	15	22.1
Environmental	1	5.9	-	-	1	1.5
Leisure related	-	-	1	3.6	1	1.5
Unlikely	12	70.6	6	21.4	21	30.9
Overall	20	100	38	100	80	100

The median survival of pericardial MM was 2.5 (SE 1.0) months; 6.8 (SE 0.6) for females and 1.4 (SE 0.6) for males. For TVT MM cases, the median survival was 33.0 (SE 7.8) months. Results of Cox’s proportional hazard model for pericardial MM survival showed that older subjects (>75 years) had a hazard risk equal to 3.52 (95% CI 1.45–8.51), with respect to subjects <65 years (table 2a and figure 1a). Pericardial mesothelioma cases with sarcomatoid morphology presented a risk of 1.42 (95% CI 0.53–3.80, P-value 0.6), but this finding, although established in pleural and peritoneal mesothelioma, has to be considered with extreme caution due to the small sample size. Similar findings have been obtained for TVT MM, with a more favorable prognosis for younger patients, (table 2b and figure 1b). Asbestos exposure has been assessed for 113 out of 138 (81.9%) MM cases included in this study, after a direct (56/113=49.6%) or indirect (to a next of kin) interview (57/113=50.5%) (table 1). Occupational exposure to asbestos (definite, probable or possible) was present for 61.9% of interviewed subjects (70/113), with differences by gender and site (23.6% in females pericardial MM, 75.0% in males pericardial MM and 66.2% in TVT MM). The economic sectors more frequently associated with asbestos exposure were construction, steel mills, metal-working industry, textile industry and agriculture. Asbestos exposure in this last sector in Italy has been previously described in ReNaM reports, due mainly to the maintenance of rural buildings containing asbestos.

**Table 2a:** Cox proportional hazards regression for prognostic factors in survival. Relative risk (RR) and 95% confidence interval (CI) by gender, age at diagnosis, period of incidence, diagnostic certainty and morphology. Pericardial malignant mesothelioma (MM), Italy, 1993–2015 (N=58). [NOS=not otherwise specified.]

	RR	95% CI	P-value
Gender			
Men	1	-	
Women	0.53	0.27-1.02	0.06
Age at diagnosis (years)			
0–64	1	-	
65–74	1.28	0.66-2.49	0.46
≥75	3.52	1.45-8.51	<0.05
Period of incidence			
1993–2003	1	-	
2004–2015	0.76	0.42-1.39	0.38
Diagnostic certainty			
MM definite	1	-	
MM probable or possible	1.50	0.68-3.31	0.32
Morphology			
Epithelioid	1	-	">
Biphasic	0.98	0.43-2.24	0.96
Sarcomatoid	1.42	0.53-3.80	0.49
MM NOS	0.74	0.35-1.59	0.44
Not available	1.09	0.36-3.30	0.87

**Figure 1a:**
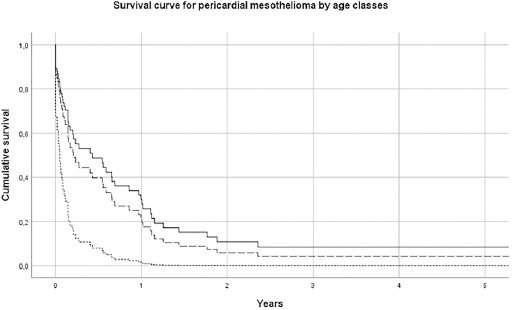
Survival curve by age-class for pericardial mesothelioma. Italy, 1993–2015 (N=58). [Age classes: ≤64 years = solid line; 65–74 years = long dashed line; ≥75 years = short dashed line.]

**Table 2b:** Cox proportional hazards regression for prognostic factors in survival. Relative risk (RR) and 95% confidence interval (CI) by age at diagnosis, period of incidence and morphology. Tunica vaginalis testis malignant mesothelioma (MM), Italy, 1993–2015 (N=80). [NOS=not otherwise specified.]

	RR	95% CI	P-value
Age at diagnosis (years)			
0–64	1		
65–74	3.31	1.40–7.84	<0.05
≥75	4.93	2.26–10.80	<0.05
Period of incidence			
993–2003	1		
2004–2015	1.00	0.55–1.82	0.99
Diagnostic certainty			
MM definite	1		
MM probable or possible	1.23	0.39–3.84	0.73
Morphology			
Epithelioid	1		
Biphasic	0.92	0.43–1.97	0.83
Sarcomatoid	0.34	0.08–1.42	0.14
MM NOS	0.99	0.49–1.99	0.97

**Figure 1b:**
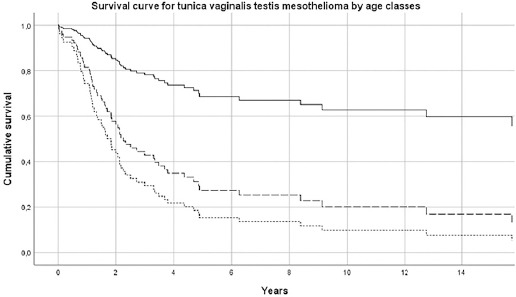
Survival curve by age-class for tunica vaginalis testis mesothelioma. Italy, 1993–2015 (N=80). [Age classes: ≤64 years = solid line; 65–74 years = long dashed line; ≥75 years = short dashed line.]

### Case–control study

We included 45 cases (28 men, 17 women) of pericardial MM and 68 cases of TVT MM. There were 929 controls (593 men, 336 women). There were 74 subjects exposed to asbestos (27 pericardial MM and 47 TVT MM) and occupational exposure was largely predominant (70 MM cases). Considering pericardial MM, an overall significant risk was found for occupational asbestos exposure (OR 3.68, 95% CI 1.85–7.31), with relevant gender difference (OR 5.52, 95% CI 2.14–14.2 and OR 1.99, 95% CI 0.60–6.63 in men and women, respectively) [table 3]. The low number of non-occupationally exposed cases (two cases) leads to statistically unstable risk estimates. The OR of occupational exposure to asbestos for TVT MM risk was 3.42 (95% CI 1.93–6.04). The overall OR of pericardial MM for asbestos-exposed subjects (either occupational or non-occupational) were 1.79 (95% CI 0.95–3.34) and 2.25 (95% CI 1.30–3.90) for pericardial and TVT MM, respectively (data not shown). Sensitivity analyses gave comparable results (tables S7–7), whereas the analyses by economic sectors did not show specific risks.

**Table 3:** Odds ratios (OR) and 95% confidence intervals (CI) of pericardial and tunica vaginalis testis mesothelioma by asbestos exposure, from conditional logistic regression models (risk set: age category: adjusted for gender), Italian national mesothelioma registry (ReNaM), 1993-2015. [NC=not calculated.]

Asbestos exposure	Cases	Controls	OR	95% CI
**Pericardium MM (women)**	17	336		
Occupational	4	37	1.99	0.60-6.63
Occupational (definite/probable)	1	16	1.23	0.15-10.3
Occupational (possible)	3	21	2.55	0.65-10.0
Non-occupational	1	101	0.18	0.02-1.41
Familial	0	46	NC	
Environmental	1	39	0.50	0.06-4.08
Leisure related	0	16	NC	
Unlikely	12	198	1.00	Reference
**Pericardium MM, men**	28	593		
Occupational	21	208	5.52	2.14-14.2
Occupational (definite/probable)	12	125	5.83	2.06-16.5
Occupational (possible)	9	83	5.45	1.86-16.0
Non-occupational	1	102	0.47	0.06-3.93
Familial	0	42	NC	
Environmental	0	46	NC	
Leisure related	1	14	5.33	0.58-49.4
Unlikely	6	283	1.00	Reference
**Pericardium MM, women and men**	45	929		
Occupational	25	245	3.68	1.85-7.31
Occupational (definite/probable)	13	141	3.50	1.56-7.84
Occupational (possible)	12	104	3.90	1.76-8.66
Non-occupational	2	203	0.28	0.06-1.21
Familial	0	88	NC	
Environmental	1	85	0.36	0.05-2.77
Leisure related	1	30	1.01	0.13-7.95
Unlikely	18	481	1.00	Reference
**Tunica vaginalis testis MM**	68	593		
Occupational	45	208	3.42	1.93-6.04
Occupational (definite/probable)	30	125	4.19	2.22-7.90
Occupational (possible)	15	83	2.57	1.25-5.31
Non-occupational	2	102	0.27	0.06-1.18
Familial	1	42	0.31	0.04-2.38
Environmental	0	46	NC	
Leisure related	1	14	1.35	0.16-11.3
Unlikely	21	283	1.00	Reference

## Discussion

As a legacy of the massive use of asbestos until the 1992 ban, Italy is today one of the countries most affected by asbestos-related diseases. Thanks to a long-term epidemiological surveillance of MM incidence, which covers the Italian population almost completely, our study provides – to our knowledge for the first time – a comprehensive nationwide picture of pericardial and TVT MM epidemiology. Furthermore, this is the first analytical epidemiological study to evaluate the risk related to asbestos exposure for these rare diseases, showing a significant association between occupational asbestos exposure and both pericardial and TVT MM incidence, supporting the evidence that asbestos cause MM in all anatomical sites.

Pericardial and TVT MM are such extremely rare diseases that incident rates are seldom estimated at the population level. Such a low incidence level prevents exercises of correlation between past asbestos consumption, as a proxy of exposure, and incidence, similar to those performed for pleural and pericardial mesothelioma (25, 26). In asbestos-exposed cohorts, disease rarity prevents any meaningful analysis. Even at the national level, it is very difficult to discuss the epidemiology of pericardial and TVT MM. In Italy, previous cases reports by specialized cancer registries have been published for pericardial and TVT MM at regional (11, 27, 28) and national level (29).

Potential risk factors for pericardial MM, other than asbestos exposure, have been suggested in some case reports or literature reviews: therapeutic ionizing radiation exposure, smoking, chemotherapeutic treatment and history of cardiovascular diseases (11, 16, 30, 31), but no epidemiological study has ever tested these hypotheses. Hydrocele, inguinal hernia or infection and trauma, ionizing radiation and tobacco smoking have been supposed as potential risk factors for TVT MM without experimental or epidemiological confirmations (16, 32). Most of the studies do not contain any information about asbestos exposure. A review of 27 case reports on pericardial MM showned that a third of the patients were exposed to asbestos (33); Guney and colleagues (34) reviewed 74 cases of TVT MM and found that 34.2% of patients presented a history of asbestos exposure. Recently, the need to include the assessment of ionizing radiation or radiotherapy for MM cases registered by ReNaM has been discussed according to the putative role in mesothelioma risk (35).

The main strength of this study is the presence in Italy of a systematic active search of MM over the whole national territory, with standard criteria for case identification, diagnosis classification and evaluation of the occupational, environmental and familial history of affected people, obtained by the means of a structured individual questionnaire. The temporal and territorial extent of the Italian surveillance system comprises >1000 million of person-years of observations. Mesothelioma epidemiological surveillance systems, comparable to Italian experience for information completeness, exposure assessment and territorial coverage, are rare and – to the best of our knowledge – currently present only in Australia, France and South Korea (36–39). Notwithstanding the old age at diagnosis and the large period of recruitment for pericardial and TVT MM cases, the majority of diagnoses were confirmed by histology (78% and 95% respectively). The completeness and quality of diagnosis in ReNaM have been confirmed by comparison with the Italian cancer registries (40). The interview rate was 85% for TVT MM and 78% for pericardial MM, even despite the poor prognosis. All cases and controls included in case–control study have been interviewed using the ReNaM questionnaire, occupational histories were coded with the same classifications of industry and occupation, and exposure was assessed according to the same protocol.

Some limitations of this study have to be considered. Cases of pericardial and TVT MM used in this study have been extracted by ReNaM archives. ReNaM methods in detecting, classifying and coding MM cases have been repeatedly published in the literature (20, 41). ReNaM is collecting MM cases across Italy, but the activity of its regional operating centers did not begin at the same time and this could have biased our study given the inhomogeneous territorial distribution of industrial and natural sources of asbestos exposure. Exposure assessment was qualitative, and the ability to identify the modalities of asbestos exposure effectively was not fully consistent among regional registries despite the use of a shared structured questionnaire. The percentage of collected exposure histories varied between 45–95% among regions. Furthermore, the possible lack of homogeneity among COR in classifying and coding diagnoses and exposures (according to the national guidelines) has to be considered. Finally, as generally is the case with specialized registries, potential over-reporting could be a concern for ReNaM. Limitations of the case–control study is that control samples refer to previous studies conducted between 2012 and 2015 with a temporal mismatch with MM cases (1993–2015). The incomplete time coverage of controls was partially compensated by being age-matched and the fact that we considered lifetime asbestos exposure, which is likely to be fairly constant after the 1992 asbestos ban in Italy, even if the amount of asbestos-containing materials removed after the ban was consistent and could introduce a bias that is difficult to assess. Furthermore, lifetime occupational asbestos exposure among male cases and controls showed little variation except for those born on or after 1960. Sensitivity analyses with temporal restrictions (only period 2000–2015 and only subjects born before 1950) were in line with the results of the main analysis (tables S5 and S6). Geographical coverage of controls was also incomplete, with controls enrolled only from Apulia, Lombardy, Piedmont, Tuscany, Veneto and Emilia-Romagna regions. These regions were (and still are) among the most industrialized areas in Italy, and it is highly plausible that lifetime asbestos exposure is higher in the population living in these regions than in other regions that did not provide controls. Case–control sensitivity analyses, restricted to the six regions which enrolled control subjects, yielded ORs that were comparable with those found in the main analysis. Finally, the qualitative reconstruction of exposure and the contemporary use of chrysotile and amphiboles in many occupational settings in Italy did not allow for any separate analysis between different varieties of asbestos fibers.

In conclusion, this study provided further evidence of the extreme rarity of pericardial and TVT MM, with a mean annual incidence rates in Italy <1 case per 10 million person-years. Survival analyses confirmed the very poor prognosis for pericardial MM and the prognostic role of age for both pericardial and TVT MM (more favorable for younger patients). Finally, for the first time, our analytical epidemiological study showed an association between asbestos exposure and pericardial and TVT MM risk, supporting the causal role of asbestos for MM of all anatomical sites.

## Supplementary material


