# Supplementary material for: Association between asbestos exposure and pericardial and tunica vaginalis testis malignant mesothelioma: a case–control study and epidemiological remarks
Source: Scand J Work Environ Health. 2020 Oct 30;46(6):609–17. doi: 10.5271/sjweh.3895 (PMC7737812; doi:10.5271/sjweh.3895)
Supplement: Supplementary file 1 [file SJWEH-46-609-S001.pdf]

Association between asbestos exposure and pericardial and tunica vaginalis testis malignant mesothelioma: a case–control study and epidemiological remarks [1]

by Alessandro Marinaccio, MSc,[2] Dario Consonni, PhD, Carolina Mensi, PhD, Dario Mirabelli, MD, Enrica Migliore, MSc, Corrado Magnani, MD, Davide Di Marzio, BSc, Valerio Gennaro, PhD, Guido Mazzoleni, MD, Paolo Girardi, PhD, Corrado -Negro, PhD, Antonio Romanelli, MD, Elisabetta Chellini, MD, Iolanda Grappasonni, PhD, Gabriella Madeo, MD, Elisa -Romeo, MD, Valeria Ascoli, PhD, Francesco Carrozza, MD, Italo Francesco Angelillo, PhD, Domenica Cavone, MSc, Rosario Tumino, MD, Massimo Melis, MD, Stefania Curti, PhD, Giovanni Brandi, MD, Stefano Mattioli, MD, Sergio Iavicoli, PhD, ReNaM Working Group *

1. *Supplementary material*
2. *Correspondence to: Alessandro Marinaccio, Epidemiology Unit, Occupational and Environmental Medicine, Epidemiology and Hygiene Department, INAIL, Via Stefano Gradi 55, 00143 Rome, Italy. [E-mail: a.marinaccio@inail.it]*

Table S1. Person years of observation (thousands) by region and year of incidence. Italian mesothelioma registry (ReNaM), Italy, 1993-2015

	1993	1994	1995	1996	1997	1998	1999	2000	2001	2002	2003	2004	2005	2006-2015	1993-2015
Piemonte															98,906
Valle d'Aosta															1,982
Liguria															31,871
Lombardia															151,604
P.A. Bolzano															5,447
P.A. Trento															10,348
Veneto															106,519
Friuli-Venezia Giulia															25,158
Emilia-Romagna															82,854
Toscana															82,275
Marche															30,062
Umbria															8,896
Lazio															81,641
Abruzzo															20,834
Campania															86,330
Puglia															92,803
Basilicata															12,438
Sicilia															94,740
Total Men	7,875	7,870	8,950	12,295	12,295	14,728	14,725	19,739	25,005	25023	25,192	25,481	26,013	271,237	496,428
Total Women	8,364	8,363	9,521	13,132	13,139	15,726	15,732	21,082	26,696	26721	26,861	27,112	27,608	288,221	528,278
Total	16,239	16,233	18,471	25,427	25,434	30,454	30,457	40,821	51,701	51744	52,053	52,593	53,621	559,458	1,024,706

Table S2. Raw and standardized incidence rates of pericardial and tunica vaginalis testis mesothelioma by gender and period of incidence (Italian, European and world standard population, per million person-years).

	Incidence rates (per milion person-years)	Pericardial MM		Tunica vaginalis testis MM
		Women	Men	
**1993-2015**	Incident cases	20	38	80
	Raw rates	0.038	0.077	0.161
	Std rates (ITA)	0.032	0.071	0.155
	Std rates (EUR)	0.036	0.080	0.178
	Std rates (WORLD)	0.023	0.049	0.095
**1993-1997**	Incident cases	3	5	8
	Raw rates	0.057	0.101	0.162
	Std rates (ITA)	0.040	0.095	0.166
	Std rates (EUR)	0.053	0.119	0.182
	Std rates (WORLD)	0.026	0.055	0.114
**1998-2003**	Incident cases	9	12	23
	Raw rates	0.068	0.096	0.185
	Std rates (ITA)	0.056	0.093	0.186
	Std rates (EUR)	0.065	0.101	0.211
	Std rates (WORLD)	0.039	0.067	0.116
**2004-2009**	Incident cases	2	11	24
	Raw rates	0.012	0.069	0.151
	Std rates (ITA)	0.013	0.064	0.145
	Std rates (EUR)	0.013	0.071	0.167
	Std rates (WORLD)	0.010	0.050	0.089
**2010-2015**	Incident cases	6	10	25
	Raw rates	0.034	0.061	0.153
	Std rates (ITA)	0.029	0.053	0.137
	Std rates (EUR)	0.032	0.061	0.161
	Std rates (WORLD)	0.031	0.033	0.078

Table S3. Characteristics of control subjects.

	Men		Women		Total	
	N	%	N	%	N	%
**Total**	593	100	336	100	929	100
**Region (Study)**						
Apulia (MISEM)	58	9.8	14	4.2	72	7.8
Emilia-Romagna (CARA)	103	17.4	108	32.1	211	22.7
Lombardy (MISEM)	141	23.8	62	18.5	203	21.8
Piedmont (MISEM)	159	26.8	108	32.1	267	28.7
Tuscany (MISEM)	26	4.4	7	2.1	33	3.6
Veneto (MISEM)	106	17.9	37	11.0	143	15.4
**Age (years)**						
<55	78	13.2	63	18.8	141	15.2
55-59	52	8.8	25	7.4	77	8.3
60-64	62	10.5	37	11.0	99	10.7
65-69	143	24.1	61	18.2	204	22.0
70-74	121	20.4	56	16.7	177	19.0
75-79	72	12.1	35	10.4	107	11.5
80+	65	11.0	59	17.6	124	13.3
**Year of birth**						
1914-1929	26	4.4	28	8.3	54	5.8
1930-1939	142	23.9	78	23.2	220	23.7
1940-1949	249	42.0	114	33.9	363	39.1
1950-1959	103	17.4	51	15.2	154	16.6
1960-1992	73	12.3	65	19.4	138	14.8

Table S4. Odds ratios (OR) and 95% confidence intervals (CI) of pericardial and tunica vaginalis testis mesothelioma by asbestos exposure, from conditional logistic regression models (risk set: age category: adjusted for region and gender), Italian national mesothelioma registry (ReNaM), 1993-2015. Analysis restricted to regions which enrolled control subjects.

**Asbestos exposure**	**Cases**	**Controls**	**OR**	**95% CI**
**Pericardium MM, women**	15	336		
Occupational	4	37	3.13	0.84-11.7
Occupational (definite/probable)	1	16	1.84	0.19-17.5
Occupational (possible)	3	21	4.07	0.90-18.3
Non-occupational	0	101	NC	
Familial	0	46	NC	
Environmental	0	39	NC	
Leisure related	0	16	NC	
Unlikely	11	198	1.00	Reference
**Pericardium MM, men**	22	593		
Occupational	17	208	7.99	2.50-25.5
Occupational (definite/probable)	9	125	7.90	2.22-28.2
Occupational (possible)	8	83	8.57	2.35-31.3
Non-occupational	1	102	0.83	0.09-7.99
Familial	0	42	NC	
Environmental	0	46	NC	
Leisure related	1	14	8.34	0.78-88.7
Unlikely	4	283	1.00	Reference
**Pericardium MM, women and men**	37	929		
Occupational	21	245	4.38	2.03-9.46
Occupational (definite/probable)	10	141	3.94	1.57-9.91
Occupational (possible)	11	104	4.87	2.03-11.7
Non-occupational	1	203	0.21	0.03-1.71
Familial	0	88	NC	
Environmental	0	85	NC	
Leisure related	1	30	1.39	0.16-11.9
Unlikely	15	481	1.00	Reference
**Tunica vaginalis testis MM**	55	593		
Occupational	38	208	5.23	2.71-10.1
Occupational (definite/probable)	28	125	7.03	3.40-14.6
Occupational (possible)	10	83	3.18	1.32-7.62
Non-occupational	2	102	0.79	0.17-3.69
Familial	1	42	0.59	0.07-4.82
Environmental	0	46	NC	
Leisure related	1	14	2.97	0.33-26.3
Unlikely	15	283	1.00	Reference

Abbreviations: NC, not calculated

Table S5. Odds ratios (OR) and 95% confidence intervals (CI) of pericardial and tunica vaginalis testis mesothelioma by asbestos exposure, from conditional logistic regression models (risk set: age category: adjusted for region and gender), Italian national mesothelioma registry (ReNaM), 1993-2015. Analysis restricted to cases with definite diagnosis.

**Asbestos exposure**	**Cases**	**Controls**	**OR**	**95% CI**
**Pericardium MM, women**	13	336		
Occupational	3	37	1.97	0.49-7.86
Occupational (definite/probable)	1	16	1.62	0.18-14.3
Occupational (possible)	2	21	2.26	0.44-11.7
Non-occupational	1	101	0.24	0.03-1.94
Familial	0	46	NC	
Environmental	1	39	0.77	0.09-6.53
Leisure related	0	16	NC	
Unlikely	9	198	1.00	Reference
**Pericardium MM, men**	22	593		
Occupational	16	208	4.44	1.66-11.9
Occupational (definite/probable)	9	125	4.65	1.55-14.0
Occupational (possible)	7	83	4.23	1.36-13.2
Non-occupational	0	102	NC	
Familial	0	42	NC	
Environmental	0	46	NC	
Leisure related	0	14	NC	
Unlikely	6	283	1.00	Reference
**Pericardium MM, women and men**	35	929		
Occupational	19	245	3.42	1.59-7.35
Occupational (definite/probable)	10	141	3.33	1.35-8.23
Occupational (possible)	9	104	3.55	1.45-8.73
Non-occupational	1	203	0.17	0.02-1.32
Familial	0	88	NC	
Environmental	1	85	0.49	0.06-3.78
Leisure related	0	30	NC	
Unlikely	15	481	1.00	Reference
**Tunica vaginalis testis MM**	64	593		
Occupational	44	208	4.05	2.22-7.38
Occupational (definite/probable)	30	125	5.15	2.64-10.0
Occupational (possible)	14	83	2.89	1.35-6.20
Non-occupational	2	102	0.32	0.07-1.41
Familial	1	42	0.35	0.04-2.74
Environmental	0	46	NC	
Leisure related	1	14	1.71	0.20-14.5
Unlikely	18	283	1.00	Reference

Abbreviations: NC, not calculated

Table S6. Odds ratios (OR) and 95% confidence intervals (CI) of pericardial and tunica vaginalis testis mesothelioma by asbestos exposure, from conditional logistic regression models (risk set: age category: adjusted for gender), Italian national mesothelioma registry (ReNaM), 1993-2015. Analysis restricted to years 2000-2015.

**Asbestos exposure**	**Cases**	**Controls**	**OR**	**95% CI**
**Pericardium MM, women**	13	336		
Occupational	3	37	2.22	0.55-8.98
Occupational (definite/probable)	0	16	NC	
Occupational (possible)	3	21	3.50	0.83-14.7
Non-occupational	1	101	0.28	0.03-2.24
Familial	0	46	NC	
Environmental	1	39	0.81	0.10-6.95
Leisure related	0	16	NC	
Unlikely	9	198	1.00	Reference
**Pericardium MM, men**	23	593		
Occupational	16	208	4.17	1.57-11.1
Occupational (definite/probable)	10	125	4.82	1.65-14.1
Occupational (possible)	6	83	3.65	1.13-11.8
Non-occupational	1	102	0.46	0.06-3.90
Familial	0	42	NC	
Environmental	0	46	NC	
Leisure related	1	14	5.14	0.55-47.8
Unlikely	6	283	1.00	Reference
**Pericardium MM, women and men**	36	929		
Occupational	19	245	3.36	1.57-7.19
Occupational (definite/probable)	10	141	3.26	1.33-7.99
Occupational (possible)	9	104	3.52	1.44-8.57
Non-occupational	2	203	0.35	0.08-1.53
Familial	0	88	NC	
Environmental	1	85	0.45	0.06-3.49
Leisure related	1	30	1.32	0.17-10.5
Unlikely	15	481	1.00	Reference
**Tunica vaginalis testis MM**	61	593		
Occupational	39	208	3.09	1.71-5.57
Occupational (definite/probable)	28	125	4.09	2.13-7.86
Occupational (possible)	11	83	1.98	0.89-4.37
Non-occupational	2	102	0.28	0.06-1.24
Familial	1	42	0.32	0.04-2.51
Environmental	0	46	NC	
Leisure related	1	14	1.36	0.16-11.5
Unlikely	20	283	1.00	Reference

Abbreviations: NC, not calculated

Table S7. Odds ratios (OR) and 95% confidence intervals (CI) of pericardial and tunica vaginalis testis mesothelioma by asbestos exposure, from conditional logistic regression models (risk set: age category: adjusted for gender), Italian national mesothelioma registry (ReNaM), 1993-2015. Analysis restricted to subjects born before 1950.

Asbestos exposure	Cases	Controls	OR	95% CI
**Pericardium MM, women**	12	220		
Occupational	4	29	2.40	0.63-9.10
Occupational (definite/probable)	1	13	1.70	0.19-15.3
Occupational (possible)	3	16	2.84	0.61-13.2
Non-occupational	1	73	0.26	0.03-2.17
Familial	0	31	NC	
Environmental	1	31	0.66	0.08-5.68
Leisure related	0	11	NC	
Unlikely	7	118	1.00	Reference
**Pericardium MM, men**	21	417		
Occupational	17	157	6.70	1.91-23.6
Occupational (definite/probable)	9	97	6.31	1.65-24.1
Occupational (possible)	8	60	7.53	1.88-30.2
Non-occupational	1	68	0.97	0.10-9.49
Familial	0	23	NC	
Environmental	0	34	NC	
Leisure related	1	11	7.07	0.67-75.0
Unlikely	3	192	1.00	Reference
**Pericardium MM, women and men**	33	637		
Occupational	21	186	4.14	1.78-9.59
Occupational (definite/probable)	10	110	3.59	1.36-9.46
Occupational (possible)	11	76	4.83	1.87-12.5
Non-occupational	2	141	0.45	0.10-2.13
Familial	0	54	NC	
Environmental	1	65	0.53	0.07-4.26
Leisure related	1	22	1.45	0.17-12.1
Unlikely	10	310	1.00	Reference
**Tunica vaginalis testis MM**	51	417		
Occupational	36	157	3.28	1.66-6.46
Occupational (definite/probable)	26	97	3.97	1.94-8.16
Occupational (possible)	10	60	2.20	0.89-5.45
Non-occupational	2	68	0.43	0.09-1.96
Familial	1	23	0.74	0.09-5.96
Environmental	0	34	NC	
Leisure related	1	11	1.30	0.15-11.2
Unlikely	13	192	1.00	Reference

Abbreviations: NC, not calculated
